# Iranian women’s perception on the determinants of birth experience: a qualitative study

**DOI:** 10.1186/s12884-022-05078-z

**Published:** 2022-10-05

**Authors:** Mojgan Mirghafourvand, Shahla Meedya, Eesa Mohammadi, Sakineh Mohammad-Alizadeh-Charandabi, Mohammad Asghari Jafarabadi, Solmaz Ghanbari-Homaie

**Affiliations:** 1 Social determinants of Health Research Center, Tabriz University of Medical sciences, Tabriz, Iran; 2 School of Nursing and Midwifery, Faculty of Health Sciences, Australian Catholic University, Sydney, Australia; 3 Department of Nursing, School of Medicine, Tarbiat Modares University, Tehran, Iran; 4 Cabrini Research, Cabrini Health, 3144 Melbourne, VIC Australia; 5 School of Public Health and Preventative Medicine, Faculty of Medicine, Nursing and Health Sciences, Monash University, 3800 Melbourne, VIC Australia; 6 Road Traffic Injury Research Center, Tabriz University of Medical Sciences, Tabriz, Iran; 7 Department of Midwifery, Faculty of Nursing and Midwifery, Tabriz University of Medical Sciences, Tabriz, Iran

**Keywords:** Primiparous women, Sense of control, Sense of safety, Support, Woman centred-care

## Abstract

**Background:**

The prevalence of cesarean birth in Iran is very high. Having a negative childbirth experience is one of the reasons that primiparous women provide to prefer caesarean birth over a vaginal birth. This study is aimed to understand women’s perspective on what determines a positive or negative birth experience for them.

**Methods:**

This qualitative study is a part a mixed method study that was conducted among primiparous women with a previous vaginal birth experience. The purpose of the main study was to develop a guideline based on Iranian primiparous women’s birth experiences. The quantitative phase of the study was a cross-sectional study where women’s childbirth experiences was measured in a survey via the Childbirth Experience Questionnaire-2. In the qualitative part of the study, women were invited for an in-depth interview via a random stratified sampling method based on their childbirth mean score (women with 10% of the upper bound score which indicated a positive birth experience and 10% of the lower bound indicating negative birth experience, n = 17). Conventional content analysis was used for data analysis.

**Results:**

We extracted three main themes: (a) “Internal control”, (b) “External control”, and (c) “Support”. Possessing internal control, having a balanced external control to feel cared and feeling supported were the main reasons for women to feel positive about their birth experiences. Whereas, loss of internal control, imbalanced external control and unsupportive environment were related to their negative childbirth experiences.

**Conclusion:**

Considering that women’s sense of control, the care and support that they receive can influence their childbirth experiences, there is a need for changing maternity policies and practices to highlight the importance of a woman-centred care to create a pleasant, respectful and positive memory for primirparous women who experience normal vaginal births.

## Background

According to the World Health Organization’s recommendations (WHO), the acceptable rate of caesarean birth ranges from 10 to 15% [1]. In Iran, the prevalence of cesarean birth is very high (48%), and it is estimated to be about 66% in some cities [2]. Although cesarean birth is known to be a safe procedure in most countries, there is no evidence that an unnecessary cesarean births can improve maternal or neonatal outcomes [1, 3–5]. On the other hand, cesarean births same as any other surgeries, have short and long-term complications such as postpartum hemorrhage, infection, low Apgar scores, birth asphyxia, delay in communication between mother and infants [1, 3–5].

Main medical reasons for unnecessary cesarean birth in Iran include (a) reduction in perinatal mortality and neonatal morbidity and (b) misconceptions about the safety of a cesarean birth over vaginal birth [6–10]. In a cross-sectional study, Iranian women’s reasons for preferring caesarian birth were related to having pervious cesarean birth, their fear towards a normal birth, having a negative childbirth experience, and physician’s suggestion [2]. Among these factors, negative birth experience is one of the outstanding reasons for the primiparous women to prefer a caesarean birth over a normal vaginal birth [2].

The birth experience is defined as a personal life event that is a combination of physiological, mental, and psychological processes [11]. Women with positive birth experiences see birth as a source of power and feel empowered and self-confident in life [12]. However, women with negative birth experiences may develop fear for their next pregnancy, postpartum depression, and post-traumatic stress disorder [13–16]. Evidence from developed countries [17–19], demonstrated that women’s birth experiences have been influenced by the following factors: (a) women’s expectations of birth, (b) their involvement in decision-making process, (c) the quality of the support they receive, (d) and their relationship with caregivers [20, 21].

Considering that human experiences in each community can be influenced by cultural, economic, and social differences [22], we conducted this study with a group of Iranian women who may have different needs compared to women who live in developed countries. The aim of the qualitative part of our study was to understand a group of Iranian women’s perspective on what they believe that determined a positive and negative birth experience for them.

The analysis of women’s birth experiences can help caregivers to better understand maternal needs and expectations in order to provide a woman-centred care [11, 23]. Based on our knowledge this is the first Iranian qualitative study that has given voice to women and let them be heard. This study reports on women’s true experiences during the birth of their first babies.

## Method

### Study design

The study protocol has been approved by the Ethics Committee of the Tabriz University of Medical Sciences, Tabriz, Iran (Code: IR.TBZMED.REC.1396.786). The informed written consent was taken from all participants, who were reassured that their identity and information would be kept confidential throughout all the implementation and publication stages of research.

This study was part of a mixed method study aiming to develop a guideline based on Iranian primiparous women’s birth experiences. The first part of the study was a cross-sectional study which 800 primiparous women. In this part, women’s birth experience was measured by Childbirth Experience Questionnaire-2 and the predictors of positive or negative birth experience were identified. In the second part which is the focus of this manuscript, we aimed to understand women’s perspective on what they believe that determined a positive and negative birth experience for them. The research methodology is described thoroughly in the study protocol [24].

### Setting

The research settings in the study included tertiary educational hospitals, private and organizational (affiliated with military organs) hospitals in Tabriz city. Tabriz is a metropolitan city and is the third largest city in Iran with Azery heritage.

### Participants

This study was conducted among primiparous women who experienced a vaginal birth, at least one to four months prior the study recruitment. The reason for choosing this timeframe was to avoid any false positive or negative birth experiences immediately after the birth due to the excitement of having a baby [25]. The eligibility criteria were healthy primiparous mothers aged 19 or more who had a vaginal birth to a full term healthy baby. Women with a history of depression or any major medical complications were excluded from the study.

### Sampling

In the quantitative part of the study, 800 women were randomly selected from the Tabriz urban and suburban health centers (n = 114). The researcher extracted and listed the eligible mothers’s names from each health center. Mothers at one to four months postpartum were randomly selected for each center using the website of www.random.org.

The women who consented to participate in the study were asked to complete Childbirth Experience Questionnaire-2 (CEQ 2.0). The CEQ 2.0 contains 23 items with four subscales (“own capacity”, “professional support”, “participation”, and “perceived safety”. The range of total score is between 1 and 4, and upper scores show more positive experience. The Persian version of the CEQ 2.0 is a valid and reliable tool [25, 26].

In the qualitative part of the study, women from the main study-cohort were invited for an in-depth interview (n = 142). A random stratified sampling was employed based on women’s positive (10% of the upper bound score) or negative (10% lower bound score) birth experience scores.

### Data collection

After consenting process, the qualitative data were collected by conducting an in-depth interview using open-ended questions such as “could you please tell me about your experience during labour and birth?”. SGH (Corresponding author), interviewed the women in any places that they felt comfortable (health center, woman’s house, hospital Ph.D.’s student room and faculty room). The interview time varied between 20 and 60 min. Although data saturation was reached after 12 interviews, we interviewed 17 women in total. Eight women with negative experiences and nine women with positive experiences. A total number of 800 mothers were recruited in the study between May and November 2018. Furthermore, interviews were conducted within six months between August and March 2019.

### Trustworthiness and rigor

“Credibility”, “transferability”, “dependability”, and “conformability” were assessed to confirm the accuracy of study results [27]. The coded texts were provided to some women for checking the accuracy of the interpretations. The data were also provided to the third (EM) and first author (MM) to confirm the consistency of the categories with the women’ statements. The research process was described in details to allow other researchers to assess the research process. The categories were checked by the rest of authors to make sure that the themes reflect the women’ voices instead of the researcher’s preconceptions or views. We used the Standards for Reporting Qualitative Research Checklist (SRQR) to write this article. Previous publications include more details on the rigor of the study [28].

### Data analysis

The data were transcribed using the verbatim transcription and then conventional content analysis was employed to analyze the data [29]. The following steps were taken during the data analysis: (a) reading repeatedly the hard copy of the printed transcript, (b) extracting the codes, (c) categorizing the codes based on their differences or similarities, (d) identifying the connections and relationships between the subcategories to organize the main categories (themes) (performed by the corresponding author (SGH) under the supervision of the third author (EM), who is an expert in qualitative research). We did not use any software. You used handwritten method for the data analysis.

## Results

In-depth individual interviews were conducted among 17 participants within six months in 2019.

All of the participants had Azari Heritage and majority of them were house-wives with tertiary education (Table 1). Forty and 102 women gain 10% of the lower bound score (negative birth experience) and 10% of the upper bound score (positive birth experience) from the CEQ, respectively.

**Table 1:** The demographic characteristic of the participants (n = 17)

Variable	n
**Age (Years)**	
18 to 25	8
26 to 30	7
31 and above	2
**Highest Education**	
Below high school	3
Completed high school	3
Tertiary	11
**Occupation**	
Housewife	14
Employee*	4
**Delivery place (Hospital)**	
Teaching	11
Military affiliated	1
Private	5

*Nurse, Teacher, Lawyer, Secretary

Three main themes extracted: (a) “Internal control”, (b) “External control”, (c) “Support”. Women who felt positive about their childbirth possessed inner control, felt respected and cared, and believed that they received the support they needed whereas, women with negative childbirth experience, did not feel that they had inner control or power; they felt unsafe and did not receive a disrespectful or supported care (Table 2).

**Table 2:** Themes and sub themes of birth experience’s determinants perceived by women

Sample quotes or Positive birth experience	Categories for Positive birth experience	Main themes (Sub themes)	Categories for Negative birth experience	Sample quotes for Negative birth experience
	**Possessing an internal control**	**Internal control**	**Loss of internal control**	
*“Now that I remember the moment when they brought the baby and put it on my chest, I wouldn’t change that juncture with nothing. It felt really good and it greatly affected my sense of satisfaction with the birth.”* *“I was always praying and begging to God for a vaginal birth. Thank God it went well.”* “*My cervix opened sooner because of the exercises I did. I searched for all of the pregnancy exercises on the Internet and did them two or three times a week. I walked for 30 minutes every morning and night. I think exercising was an important factor in making my birth easier. I returned to my daily activities five days after birth and started doing everything myself.”* *“I myself wanted to feel what a woman feels as a mother during a vaginal birth. I thought that C-section would not give me such feeling and that the mother-child relationship would be stronger after vaginal birth. I wanted to sense that feeling. I wanted to experience the labour pain.”* *“I didn’t scream at all when I felt pain. My mind was at peace. I didn’t yell. I think yelling cannot solve anything. The staff was pleased with me (she chuckles), and the doctor told me that I was really good at pain endurance”*	- Maternal and child attachment - Praying and trusting in God - Preparedness before labor - Woman’s positive attitude towards vaginal birth - Feeling of self-control	(**Sense of security and trust)** **(Sense of power)**	- Feeling of fear and worry - Feeling of powerlessness	*“Thinking that I would die, I was saying my last prayers. Everybody is afraid of vaginal birth, especially a person like me who was experiencing it for the first time.”* *“I lose my power quickly and feel tired soon. I’m not physically very strong.”*
	**Balanced external control**	**External control**	**Imbalanced external control**	
*“I took hot showers at my back, and it had a really good effect. It relieved my pains so much that I thought I could give birth to my child at that moment. It soothed my nervous system. I was really pleased with the hot shower they provided for me.”* *“It was good that I was given only one examination in the clinic by my midwife. I only had another one vaginal examination at hospital by same midwife.”* *“The room was designed for only one woman. I stayed there from the moment I felt pain until I gave birth to my child. There were no other women in my room so that I would feel embarrassed.”* *“I visited a midwife’s office for an examination. After examination, she said it was not the time. She then advised me to return home and take a hot shower. I endured most of my pain at home. We went to the hospital at 7 PM and had my son born at 8:30 PM. I am very happy with spending most of my time at home and taking a warm bath and massage by my sister.”* *“I had no bleeding and complications the morning after I gave birth. I know that you couldn’t be the same as before, but I managed to do my tasks myself and take care of my baby, which was pretty satisfactory.”* *“The presence of my sister was really pleasurable for me (she chuckles). The doctor asked me who I was comfortable with and who I wanted to be with. I said that I was fine with my sister. They asked my opinion.”*	- Satisfaction with pain relief methods - Convenience and satisfaction with the necessary interventions - Appropriate physical and psychological environment of hospital - Short stay in the labour room - Woman’s satisfaction with the childbirth outcomes - Woman’s participation in care process	**(Quality of procedural Maternity care)** **(Quality of Communicational and respectful Maternity care)**	- No use of pain relief methods - Discomfort from unnecessary interventions - Inappropriate physical and psychological environment of hospital - Maternal-neonatal complications - Not having a touch with the baby after birth - Women’s expectation and variation from normal labour and birth - Non-participation of woman and her family in care process	*“They put stitches and then they realised that the placenta remained inside the uterus. They put their hands inside my womb and after removing the placenta, they fixed my stitches, again, it was very painful.”* *“I felt pain when the doctor pushed my belly, but they said I wouldn’t be able to give birth to my child if they didn’t push. It drives me nuts whenever I remember the scene!”* *“When I entered the birth room, I felt that I was in a slaughterhouse. Because women were screaming and every table had many metal instruments including knives and scissors. I wish they were hidden somewhere.”* *“The baby went completely black. They took the baby away soon after birth. My kid did not cry, and I heard her cry after 11 days.”* *“They showed me the baby for only one moment but didn’t put it on my chest. I really wanted them to dry the baby and put it on my chest. I kept raising my head to see what my baby looked like on the other side of the room on the bed.”* *“It took three days. I stayed there from morning until night, but nothing happened. Once in a while, I had some spasms which did not feel like labour pain. I was mentally tired, perhaps because I had been in the hospital for a long time. I thought I could not give birth anymore. I was expecting someone to tell me that the waiting time will end soon or that I was going to have labour in one day. I needed hope.”* *“Most of the decisions were made by the hospital staff rather than myself. I was told in the hospital that since it was public, I had to have a vaginal birth. My husband was willing to pay the cost of a C-section, but they said it was not possible because it was a public hospital. We were not aware of this. I told them I wouldn’t have gone there if they had told us in the first place.”*
	**Receiving support**	**Support**	**Not receiving support**	
*“I’m very pleased with my husband, whom I love very much. During pregnancy, I wanted him to be with me all the time. The fact that I love my husband had a very good effect on me. I was happy with my labour because I loved my husband so much that I wanted to have a baby with him. My husband’s kindness, affection, and love had a positive effect on me, so I am happy with my labour and love my son.”* *“I really didn’t like anybody but my midwife to be with me at the time of birth. I needed specialised help which could only be provided by a midwife. My midwife was not accompanying me just for money. I think she personally liked to help. She had good manners and did whatever I wanted her to do. I spoke to her harshly when I had pain, but she didn’t respond badly.”* *“I’m very pleased with my husband, whom I love very much. During pregnancy, I wanted him to be with me all the time. The fact that I love my husband had a very good effect on me. I was happy with my labour because I loved my husband so much that I wanted to have a baby with him. My husband’s kindness, affection, and love had a positive effect on me, so I am happy with my labour and love my son.”*	- Receiving professional support - Preserving personal dignity by care provider - Receiving family support	**(Professional support)** **(Family support)**	- Disrespect and offensive behavior - Lack of support in meeting individual needs and preferences - Lack of support about labour and birth options - Negative attitude of the physicians towards vaginal birth	*“After the birth, they took me for the ultrasound scan. I had a urinary catheter. The caregiver pushed the bed so fast that it suddenly hit the wall, and the tube was broken. I became very sad and ashamed. I may not be able to verbally express the treatment I received at the hospital. They treated us as if those giving childbirth were not human.”* *“The caregivers were always writing reports and looking at their computers. They were just focused on writing their reports.”* *“It was my first childbirth experience. I had literally no information about it. Neither did I read about it before, nor did anybody tell me about it.”* *“There is not a good attitude towards vaginal birth now among specialists at hospitals. My doctor asked me why I chose vaginal birth while criticising me. He asked about my job and that of my husband and told us we had the financial status to choose C-section. I told him that it was not because of the financial problems that I had chosen vaginal birth.”*

### Internal control

Internal control had two main subthemes: (a) “sense of safety and trust”, and (b) “sense of power”.

The *“Sense of safety and trust”* were identified as part of “Internal control”. Women with positive birth experience reported that they felt in ease when they were well prepared, been positive about vaginal birth and had a chance to hold their babies and experience skin- to skin contact. According to all participants, the moment of skin contact with the baby was a unique feeling that gave then the best inner satisfaction and pure love, peace and sense of safety. Women considered that moment to be an important factor in having a positive experience.

*“Now that I remember when they brought the baby and put it on my chest, I wouldn’t change that moment with nothing. It felt really good and it greatly affected my sense of satisfaction with the birth.” (Participant 14).*

In contrast, women who were not well prepared, heard horror stories about vaginal birth and did not have a change to hold their babies felt lost, full of fear, insecure and worried.

*“Thinking that I would die, I was saying my last prayers. Everybody is afraid of vaginal birth, especially a person like me who was experiencing it for the first time.” (Participant 15).*

*“They showed me the baby for only one moment but didn’t put it on my chest. I really wanted them to dry the baby and put it on my chest. I kept raising my head to see what my baby looked like on the other side of the room on the bed.” (Participant 1).*

The “*Sense of power”* was extracted from data when women connected themselves to God and trust him through their pray.

*“I was always praying and begging to God for a vaginal birth. Thank God it went well.” (Participant 14).*

Women with sense of motherhood also reported positive childbirth experiences.

*“I myself wanted to feel what a woman feels as a mother during a vaginal birth. I thought that C-section would not give me such feeling and that the mother-child relationship would be stronger after vaginal birth. I wanted to sense that feeling. I wanted to experience the labour pain.” (Participant 10).*

Women who experienced a negative childbirth experience, repeatedly reported to feel powerless and helpless while they felt they had no sense of control during contractions. The feeling of powerless was reported under “External control” when the health care professionals made the decisions for women without engaging them in the conversation or when women felt that they were treated disrespectfully.

### External control

The main theme of *“External control”* had two subthemes: a) the “quality of procedural maternity care”, and “the quality of the communicational and respectful maternity care”.

The “quality of procedural maternity care” was very important factor for women when they described their childbirth experiences. Women with positive childbirth experiences, reported that they felt that they received appropriate maternity care including adequate non-medical pain reliefs without having unnecessary interventions such as frequent vaginal examination or extreme pressure on their abdomen during pushing phase (frequent vaginal examinations and fundal pressure during the second stage of labour are part of Iranian route maternity care).

*“I took hot showers at my back, and it had a really good effect. It relieved my pains so much that I thought I could give birth to my child at that moment. It soothed my nervous system. I was really pleased with the hot shower they provided for me.” (Participant 3).*

*“It was good that I was given only one examination in the clinic by my midwife. I only had another one vaginal examination at hospital by the same midwife.” (Participant 5).*

Women with negative childbirth experiences, in contrary, highlighted the lack of quality in the maternity care that they received. Women felt excoriated pain when they had to go through the fundal pressure, manual removal of placenta without analgesia or any pain relief.

*“I felt pain when the doctor pushed my belly, but they said I wouldn’t be able to give birth to my child if they didn’t push. It drives me nuts whenever I remember the scene!” (Participant 8).*

*“They put stitches and then they realised that the placenta remained inside the uterus. They put their hands inside my womb and after removing the placenta, they fixed my stitches, again, it was very painful” (Participant 13).*

Entering the room with many tools and gadgets, and staying in a bed with dirty lines were other reasons for women to feel negative about their childbirth.

*“When I entered the birth room, I felt that I was in a slaughterhouse. Because women were screaming and every table had many metal instruments including knives and scissors. I wish they were hidden somewhere” (Participant 1).*

*“When they broke the amniotic sac - (she paused) -, they didn’t come to change my bed sheet. I feel disgusting even now that I’m telling you” (Participant 17).*

The “the quality of the communicational and respectful maternity care” was another major factor impacting women’s childbirth experiences. Women reported positive experience when their midwives communicated with them and treated them with respect and dignity.

*“I visited a midwife’s office for an examination. After examination, she said it was not the time. She then advised me to return home and take a hot shower. I managed most of my pain at home. We went to the hospital at 7 PM and had my son born at 8:30 PM. I am very happy with spending most of my time at home and taking a warm bath and massage by my sister” (Participant 3).*

*“I really didn’t like anybody but my midwife to be with me at the time of birth. I needed specialised help which could only be provided by a midwife. My midwife was not accompanying me just for money. I think she personally liked to help. She had good manners and did whatever I wanted her to do. I spoke to her harshly when I had pain, but she didn’t respond badly.” (Participant 5).*

Women who had negative birth experiences repeatedly reported the lack of communication, lack of their involvement in decision making and being treated with disrespect which made them feel powerless.

*“After the birth, they took me for the ultrasound scan. I had a urinary catheter. The caregiver pushed the bed so fast that it suddenly hit the wall, and the tube was broken. I became very sad and ashamed. I may not be able to verbally express the treatment I received at the hospital. They treated us as if those giving childbirth were not human.” (Participant 2).*

### Support

The theme of *“Support”* was divided into the “professional support” and “family support”. Women who perceived that they were engaged in decision making felt well supported by the health professionals and family reported positive childbirth experiences.

*“The presence of my sister was really pleasant for me (she chuckles). The doctor asked me who I was comfortable with and who I wanted to be with. I said that I was fine with my sister. They asked my opinion.”(Participant 3).*

Women with negative child birth experiences reported lack of health professional support for their preferences on childbirth.

*“There is not a good attitude towards vaginal birth now among specialists at hospitals. My doctor asked me why I chose vaginal birth while criticizing me. He asked about my job and my husband’s job and told us that we could have afforded to choose C-section. I told him that it was not because of the financial problems that I had chosen vaginal birth.” (Participant 5).*

*“The caregivers were always writing reports and looking at their computers. They were just focused on writing their reports.” (Participant 4).*

## Discussion

This is the first study in an Iranian context that employs a woman-centred approach towards women’s birth experiences. The main themes extracted from women’s birth experiences are: “Internal control”, “External control”, and “Support”. Possessing internal control, having well-balanced external control with adequate care and support linked with positive experiences whereas, the loss of internal control, imbalanced external control without receiving respectful care and support were associated with negative birth experiences. Women’s sense of safety, trust and power formed their feelings towards “Internal control”.

The findings of other studies, emphasizes the importance of women’s sense of internal control influencing their satisfaction with childbirth [21, 30]. Women’s attitude towards normal birth and the mother-baby attachment were the highlights that made women feel satisfied and positive about their birth experience. Women reported that the experience of skin-to-skin contact lessened their stress and improved their satisfaction. Skin-to skin contact can help women identify and become familiar with their babies’ reactions, reduce the baby’s crying time, facilitate baby’s sleeping, improve breastfeeding initiation [31, 32] which can lead to a positive feeling for the mothers [33].

In our study, the participants used prayers and trusting in God as a strategy to feel powerful, calm and in-control. According to Moloney, pregnancy and childbirth can be considered spiritual events due to their complicated processes [34]. An epistemological study reports that women regard childbirth as an opportunity to approach God and also a spiritual experience of transformation in the contexts of religious beliefs and rituals considered a powerful coping mechanism [35].

Fear, worry and sense of powerlessness impacted Iranian women’s birth experiences negatively which is supported in other studies [36]. Women with fear of childbirth usually skip the childbirth preparation and standard care classes [37] or sometimes they are interested in using epidural anesthesia, which can increase labour interventions leading to a sense of powerlessness and a negative birth experience [12].

Although losing control during contractions made some women feel powerfulness, the main issue was related to the “External control” and the “Support” that they received. The themes of “External control” was extracted when women reported that they received a good quality of maternity care that included procedural, communicational and respectful care. The highlights for women were about receiving pain reliefs, having less interventions and participating in decision making processes. The findings of other studies demonstrated that women would not have positive birth experiences if their expectations of pain relief were not met. Despite how women interpret labour pain, most of them expect that their pains should be managed properly [38, 39]. In Iranian culture, although birth is not recognized as a gynecological surgery method, it has greatly been affected by medical interventions. For instance, frequent vaginal examination, conducting episiotomy on primiparous women and injecting oxytocin during labour are considered routine procedures where it is not an option that women can negotiate [40]. Authoritative decision-makings by midwives and medical team can make the pregnant women feel that they are crippled in the hospital hierarchy with no power and perceive their labour as traumatic [41].

Although the influence of external control is linked to the sense of gaining or losing internal control, there is a direct correlation between birth experiences and the “*Support*” that women feel from the health professionals and family during labour and birth [42, 43]. Emotional support at the time of stress, can help labouring women feel that they were being taken care of [44]. However, most participants in our study who had a negative birth experience, believed that they did not receive respectful care or adequate support during labour. Some women had doubts in their choice and ability to undergo vaginal birth due to the lack of support or the negative attitude of their doctors, family and friends towards normal birth. Evidence demonstrated that women who are treated disrespectfully with lack of support during their childbirth, may feel ignored and lonely with a diminished sense of self [45]. Additionally, offensive care indicates a violation of women’s basic rights which can adversely affect health outcomes and women’s birth experiences [46]. Women who traumatize from birth may decide not to get pregnant again or prefer a caesarian birth for subsequent pregnancies [47].

Within a feministic visionary, some women can still find their power to protect themselves against external push towards procedures that they do not feel comfortable with [48], but this could cause a battle for some women to find a balance between their internal and external controls [49]. According to a midwifery theory called the Birth territory and midwifery guardianship, the role of midwives is to support a woman to find the balance between her inner power and external power and feel safe during her childbirth experience [50]. In this theory, women’s intrinsic power is a non-rational, spontaneous power that comes from a women’s embodied self which can be similar to the theme of “Internal control” in this study. The “External control” in our study also fits well with the extrinsic power from the theory of Birth territory [49]. The extrinsic power refers to the form of power in the environment that includes woman’s ego and the input of health professionals and support people. Midwives and any health professionals who provide a care for women during childbirth are responsible to support a woman to balance her body, mind and spirit as a whole; and optimize her psychological wellbeing where she feels safe [50]. Although there is a need for a fundamental change in Iranian maternity care towards a woman-centred care, using this midwifery theory could be a helpful to design new interventions that are tailored for women with different cultural or ethnic background when they have less power to negotiate their ways of childbirths.

## Strengths and limitations

One of the strengths of this study is that we included women who gave birth in public and private hospitals which provides a comprehensive and in-depth insight to the outcomes. Additionally, conducting interviews at least one month after childbirth can be considered as a strength, because evaluating the birth experience immediately after birth without letting women have physical and mental comfort could result in unrealistic responses. Considering that the study was conducted in one of the Iranian metropolises, this study delved into the experiences of women with Azeri heritage who make up 24% of the Iranian population. Hence, the results may not be generalized to women of other ethnicities. Also the study was focused on adult, healthy women who had a singleton full-term babies with no major abnormalities, therefore, the findings may not be comprehensive or applicable for young women or women with premature or twins birth or for women with serious medical condition.

## Implication for practice

Health professionals play an important role in supporting women during childbearing period. Therefore there is a need for changes in practice, policies and education to provide a woman-cetnred care. Women with a woman-centred care can express their needs, wishes and preferences without a fear or worries; they can be involved in decision- making process, and feel safe, cared and supported. Iranian maternity culture can only change if the policies shift the power from health professional team into the consumers by emphasizing on a respectful care where women’s values and decisions are the priority.

## Conclusion

Women’s perceived negative childbirth can be alleviated by supporting women to feel in control and safe during their childbirth. There is a need for changing maternity policies and practices to highlight the importance of well-balanced and respectful care.

## Data Availability

The datasets used and analysed during the current study are available from the corresponding author on reasonable request.
